# Green Synthesis of ZnO Nanoparticles via Ganoderma Lucidum Extract: Structural and Functional Analysis in Polymer Composites

**DOI:** 10.3390/gels10090576

**Published:** 2024-09-04

**Authors:** Ayça Can, Kadriye Kızılbey

**Affiliations:** 1 Biomedical Engineering Department, Graduate School of Natural and Applied Sciences, Acıbadem University, İstanbul 34752, Türkiye; ayca.can1@live.acibadem.edu.tr; 2 Department of Natural Sciences, Faculty of Engineering and Natural Sciences, Acıbadem University, İstanbul 34752, Türkiye

**Keywords:** eco-friendly synthesis, zinc oxide, nanoparticle morphology, polyvinyl alcohol, chitosan, composite materials, mechanical properties, hydrogel swelling

## Abstract

Metallic nanoparticles are of growing interest due to their broad applications. This study presents the green synthesis of zinc oxide (ZnO) nanoparticles (ZnNPs) using *Ganoderma Lucidum* mushroom extract, characterized by DLS, SEM, XRD, and FTIR spectroscopy analyses. The synthesis parameters, including extract/salt ratio and mixing time, significantly influenced nanoparticle yield, size, and polydispersity, with longer mixing times leading to larger, more varied particles. Specifically, the sizes of ZnNPs synthesized at a 1:1 extract/ZnCl_2_ ratio after 3 h and 24 h were 90.0 nm and 243.3 nm, with PDI values of 48.69% and 51.91%, respectively. At a 1:2 ratio, the sizes were 242.3 nm at 3 h (PDI: 43.19%) and a mixture of 1.5 nm, 117.4 nm, and 647.9 nm at 24 h (PDI: 2.72%, 10.97%, and 12.43%). Polymer films incorporating PVA, chitosan, and ZnNPs were analyzed for their morphological, spectroscopic, and mechanical properties. Chitosan reduced tensile strength and elongation due to its brittleness, while ZnNPs further increased film brittleness and structural degradation. A comparison of the tensile strength of films A and C revealed that the addition of chitosan to the PVA film resulted in an approximately 10.71% decrease in tensile strength. Similarly, the analysis of films B1 and B2 showed that the tensile strength of the B2 film decreased by 10.53%. Swelling tests showed that ZnNPs initially enhanced swelling, but excessive amounts led to reduced capacity due to aggregation. This pioneering study demonstrates the potential of *Ganoderma Lucidum* extract in nanoparticle synthesis and provides foundational insights for future research, especially in wound dressing applications.

## 1. Introduction

Metallic nanoparticles are submicron-scale particles [1] and have unique properties due to their small size, including a high surface area-to-volume ratio, specific electronic structure, and the ability to interact with light through Plasmon excitation. They can be synthesized using a variety of methods and modified with various chemical functional groups, allowing them to be conjugated with antibodies, ligands, and drugs for biomedical applications [2]. The choice of synthesis method depends on factors like desired nanoparticle size, shape, composition, and intended application. The green synthesis of metallic nanoparticles using biological methods like plants, algae, bacteria, and fungi has emerged as an eco-friendly and sustainable alternative to traditional chemical synthesis techniques. These “green” methods utilize natural reducing and stabilizing agents found in biological materials, avoiding the use of hazardous chemicals. Nanoparticles synthesized using green methods tend to be more biocompatible, as they are produced without toxic chemicals. This makes them suitable for biomedical applications [3]. They can also exhibit improved properties like antimicrobial, anticancer, photocatalytic, and agricultural activities compared to conventionally produced counterparts [4,5]. Some of the most widely studied green-synthesized metallic nanoparticles include silver, gold, iron, selenium, and copper [6]. These nanoparticles have shown promising applications in areas like antimicrobial agents, cancer treatment, environmental remediation, wound dressing, and catalysis [6,7].

Zinc is an essential element that is important for several key reasons. Zinc is necessary for the proper development and activity of immune cells, helping the body fight infections and diseases [8]. Zinc-deficient individuals experience increased susceptibility to a variety of pathogens [9]. Zinc supports the production of collagen and other proteins required for tissue regeneration. Studies have found that topical zinc oxide can enhance re-epithelialization and wound closure [10] because it is essential for the proliferation and migration of keratinocytes, fibroblasts, and endothelial cells, which are critical for wound re-epithelialization and tissue repair. Zinc has anti-inflammatory and antimicrobial properties that help reduce inflammation and prevent infection in the wound [11]. Zinc acts as an antioxidant, protecting cells from oxidative damage and supporting the immune response during wound healing [12]. Zinc oxide nanoparticles have been successfully synthesized using the extract of Ganoderma lucidum mushroom as a bio-reducing and stabilizing agent [13,14]. The use of the Ganoderma lucidum extract provides additional potential for biomedical applications, as Ganoderma is known to contain over 400 bioactive compounds with medicinal properties [14]. The nanoparticles synthesized using this extract have additional potential for biomedical applications leveraging the mushroom’s therapeutic benefits. The ZnNPs provide enhanced antibacterial and antimicrobial properties to the hydrogels due to the inherent antibacterial nature of zinc [14]. The incorporation of green synthesized ZnNPs into hydrogel matrices is a promising approach for developing advanced wound dressing materials with improved mechanical, antimicrobial, and therapeutic properties. These green synthesized ZnNPs can be incorporated into hydrogel matrices using different methods, including the in situ fabrication of NPs inside pre-formed hydrogel networks, fabrication of hydrogels employing NPs for crosslinking, and preparation of hydrogel-coated NPs [15].

Natural polymers like chitosan (Chi), alginate, and hyaluronic acid offer excellent biocompatibility and biodegradability, while synthetic polymers such as polyvinyl alcohol (PVA) and polyurethanes can be used in combination to enhance mechanical properties [16]. Hydrophilic polymers are essential for managing wound exudate and maintaining a moist environment, while antibacterial properties can be imparted through inherent characteristics (e.g., chitosan) or by incorporating antimicrobial agents [17]. The porosity and gas permeability of polymer dressings are important for facilitating oxygen and water vapor exchange, which promotes a favorable healing environment. Additionally, polymers can serve as carriers for growth factors, antimicrobials, and other therapeutic agents to enhance wound healing [18]. PVA are synthetic polymers used since the early 1930s in a wide range of industrial, commercial, medical, and food applications including resins, lacquers, surgical threads, and food-contact applications. The United States Food and Drug Administration (USFDA) allows PVA for use as an indirect food additive in products which are in contact with food. PVA is approved for use in several medical applications including transdermal patches, the preparation of jellies that dry rapidly when applied to the skin and in immediate and sustained release tablet formulations. Cross-linked polyvinyl alcohol microspheres are also used for the controlled release of oral drugs [19]. Chitosan is a linear polysaccharide which is achieved by the deacetylation of chitin, which is the second most plentiful compound in nature, after cellulose. According to the USFDA, it is a GRAS (Generally Recognized as Safe) material and therefore it has found wide pharmaceutical and biomedical applications. It is known as a bioactive compound that has shown numerous biological properties such as antitumor, immunoenhancing, antifungal, antimicrobial, antioxidant, and wound healing activities [20]. These features plus some exceptional properties such as its non-toxicity, biodegradability, biocompatibility, non-antigenicity, and low cost have led to its extensive pharmaceutical applications including biomedicine with the possibility of clinical use and uses in drug delivery systems, tissue engineering, food technology, wound healing, and the textile industry [21].

In this study, ZnNPs were synthesized by a green synthesis method. The characterization of the ZnNPs was performed using dynamic light scattering (DLS), scanning electron microscopy (SEM), X-ray diffraction (XRD), and Fourier-transform infrared (FTIR) spectrophotometers. Then ZnNPs were doped into the PVA/Chi films. PVA, Chi, and PVA/Chi films were prepared using the freeze–thaw technique. FTIR spectra were employed for the chemical analysis of the films, and SEM was used for their morphological properties. Then, swelling and tensile tests were performed to obtain the absorption capacity and mechanical strength of the films, respectively.

## 2. Results and Discussion

ZnNPs, PVA, and chitosan composites offer promising applications in drug delivery systems due to their combined properties. The hydrophilic nature of PVA and chitosan facilitates the encapsulation of hydrophilic drugs, while ZnNPs enhances the stability and controlled release profile of the drug, rendering these composites highly effective for drug delivery systems. ZnO nanoparticles, known for their natural antimicrobial properties, further increase the suitability of these composites for drug delivery systems targeting infections. A proposed study aims to optimize these composites for use in wound dressings [22]. The biocompatibility of chitosan, combined with PVA and ZnNPs, supports the development of wound dressings that not only prevent infection but also promote healing. Additionally, the swelling properties of the composite help maintain a moist environment conducive to wound healing. Also, in environmental applications, ZnNPs–PVA–Chi composites [23] have potential for water treatment research, specifically in the adsorption of heavy metals and organic pollutants from wastewater. ZnO nanoparticles have a role as photocatalysts [24], capable of degrading organic pollutants under UV light, and this enhances the effectiveness of these composites in environmental remediation.

### 2.1. Characterization of ZnNPs 

**Figure 1:**
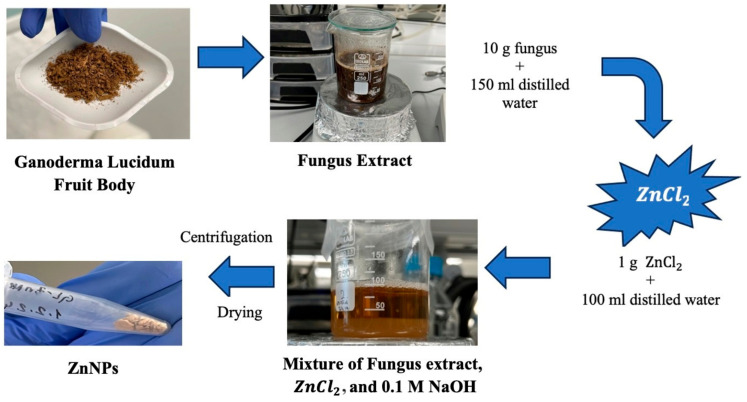
Schematic of green synthesis.

Ganoderma Lucidum mushroom extract and ZnCl_2_ salt were used to synthesize NPs by the green synthesis method. A procedure that removes solvents with the help of a centrifuge and obtains the solid phase was used, as shown in Figure 1. The green synthesis of ZnO nanoparticles leverages the reducing properties of phytochemicals found in plant extracts to transform metal precursors into nanoparticles. These naturally occurring compounds, which include antioxidants and non-toxic molecules, serve dual roles as both reducing and stabilizing agents during nanoparticle formation. Notable phytochemicals such as terpenoids, flavonoids, phenolic compounds, aldehydes, and alkaloids are pivotal in driving the reduction process. The type and concentration of these phytochemicals differ among plant extracts, which significantly impacts the efficiency of nanoparticle synthesis. Several factors—such as pH, temperature, contact time, metal salt concentration, and the specific phytochemical profile—play crucial roles in determining the synthesis, stabilization, and yield of the nanoparticles. A stabilization mechanism for metal ions in plant extracts typically involves three stages: (1) Activation Phase—the reduction and nucleation of metal ions; (2) Growth Phase—the stabilization of the formed nanoparticles; (3) Termination Phase—the final shape formation of the nanoparticles. Through the action of phytochemicals, metals like copper, silver, gold, titanium, zinc, iron, and nickel undergo transformation into their corresponding oxides. This process supports the growth and stabilization of metal ions, which subsequently combine with oxygen to form nanoparticles with distinct shapes [25]. Plants are abundant sources of biomolecules such as polyphenols, flavonoids, terpenoids, alkaloids, and saponins, all of which contribute to the green synthesis of nanoparticles by acting as reducing, capping, and stabilizing agents [26]. Ganoderma lucidum, commonly known as the Reishi mushroom, has a long history of use in traditional medicine. This mushroom is composed of non-volatile elements like ash, carbohydrates, fats, proteins, and raw fiber. Furthermore, it is rich in bioactive substances, including proteins, steroids, nucleotides, terpenoids, phenols, glycoproteins, and polysaccharides, as well as small amounts of amino acids and vitamins [27].

For the synthesis of ZnO nanoparticles specifically, 1 g of zinc chloride (≥98.00% purity) was dissolved in 100 mL of distilled water, resulting in a solution with a pH of 6.73. Various ratios of mushroom extract and zinc solution (1:1 and 1:2) were mixed for 3 h and 24 h to optimize nanoparticle formation. The mixture was subjected to ultrasonic homogenization for 4.5 min and then adjusted to pH 12 using 0.1 M NaOH. After centrifugation at 10,000 rpm for 12 min, the resulting solid phase was washed with distilled water and ethanol, dried at 60 °C, and stored at +4 °C until further use.

DLS, SEM, XRD, and FTIR were used for the characterization of ZnNPs. The yields of the produced nanoparticles were found to be in the 50–55% range based on the mushroom extract/zinc salt ratio and mixing hour (1:1—3 h, 1:1—24 h, 1:2—3 h, and 1:2—24 h). The color of the ZnNPs synthesized using Ganoderma lucidum may indeed be influenced by the organic components present in the fungal extract. It contains a variety of bioactive compounds, including proteins, polysaccharides, and secondary metabolites, which can act as reducing and stabilizing agents during the synthesis of nanoparticles [28]. These organic constituents can affect the morphology, size, and optical properties of the resulting ZnO nanoparticles. Typically, ZnO nanoparticles appear white; however, the presence of organic materials from the fungal extract can lead to color variations. The synthesis process often involves a color change that indicates the formation of nanoparticles [13]. For instance, in related studies, the color of solutions changed to beige during the synthesis of silver nanoparticles, which was indicative of successful nanoparticle formation due to the interaction with the mycelium extract. 

Green synthesis is widely recognized for its scalability and efficiency, facilitating the production of larger quantities of nanoparticles while maintaining high quality [29]. Changes in factors such as plant extract concentration, temperature, and reaction time can lead to differences in particle size and morphology, highlighting the importance of standardizing these parameters for consistent results. For instance, the application of leaf extract from *P. austroarabica* has been documented to yield zinc oxide nanoparticles with commendable reproducibility. Green synthesis not only reduces environmental impact but also provides control over particle size and morphology [30]. In this study, a rigorously optimized protocol was employed to ensure the reproducibility of the ZnNP synthesis process. To validate the consistency of nanoparticle size distribution and yield across different batches, all experiments were conducted under identical conditions using standardized protocols. Dynamic light scattering and scanning electron microscopy were utilized to evaluate the size distribution and yield of ZnNPs in each batch. The data showed that the nanoparticle sizes and their consistency were highly dependent on both the extract/ZnCl_2_ ratio and the reaction time, reflecting the reproducibility of the process. The 1:1 ratio tended to produce smaller and more uniform nanoparticles at shorter reaction times, while increasing either the ratio or the reaction time leads to larger and more variable nanoparticles. The 1:2 ratio with a 24 h reaction time was particularly notable for producing a broad distribution of particle sizes, which could be challenging for achieving consistent results in applications where uniformity is critical. Moreover, a study by Prathna et al. revealed that when Azadirachta indica leaf extract and Ag(NO)_3_ were combined, increasing the reaction time tended to produce particles with increasing size. The reaction time was varied between 30 min and 4 h to produce a change in particle size ranging from 10 to 35 nm [29,31], in agreement with this study. In summary, while the synthesis process is reproducible in the sense that it consistently produces nanoparticles under specified conditions, the resulting sizes and distributions vary significantly with changes in the reaction parameters, indicating that precise control over these variables is essential for achieving the desired nanoparticle characteristics. Additionally, the yield of ZnNP production remained within a similar range across batches, varying between 50 and 55%. These findings collectively demonstrated that the ZnNPs synthesis process has good reproducibility, consistent with the literature that zinc showed high reproducibility in an experiment performed by a green synthesis method using leaf extract [32,33]. 

To enhance the yield of ZnNPs produced through green synthesis methods using plant extracts, several optimization strategies can be considered. Increasing the concentration of the plant extract may improve the availability of reducing agents, thereby enhancing nanoparticle production. Adjusting the reaction time is also crucial, as it can provide more opportunities for nanoparticle formation, though it must be carefully balanced to avoid aggregation. Additionally, optimizing the pH of the reaction mixture and regulating the temperature can significantly impact the efficiency of nanoparticle formation. Increasing the concentration of the zinc chloride precursor can provide more zinc ions for nanoparticle synthesis, but care should be taken to prevent excessive aggregation. Improving mixing conditions to ensure uniformity in the reaction and exploring different plant extracts or additives with superior reducing properties could further boost the yield. Each of these factors should be systematically investigated to identify the optimal conditions for maximizing the yield of ZnNPs. Temperature control in the experiment can provide different yields. However, it has been shown in the literature that particle sizes will vary depending on temperature. Higher temperatures (60 °C vs. 28 °C) tend to produce larger ZnNPs with diameters in the range of 73–123 nm compared to smaller particles of 8–46 nm at lower temperatures. Elevated temperatures can enhance the aggregation of ZnNPs and increase the frequency of collisions among nucleating atoms during synthesis, leading to larger particle sizes [34]. In other experiments conducted at higher temperatures, it has been reported that optical properties and particle size also changed [35]. Reaction time stands out as one of the conditions to be selected for yield. However, it was emphasized that reaction time can be selected for the desired nanoparticle properties, noting that longer incubation times can lead to larger particle sizes and changes in morphology. In this study, the desired morphological image is consistent with the incubation time. Keeping the incubation time at 3 h resulted in the formation of round-shaped nanoparticles. However, the yield rate was shown to be 50–55%. In a study conducted with silver metal, it was reported that a 52% [36] yield was obtained in 3 h of incubation. 

**Figure 2:**
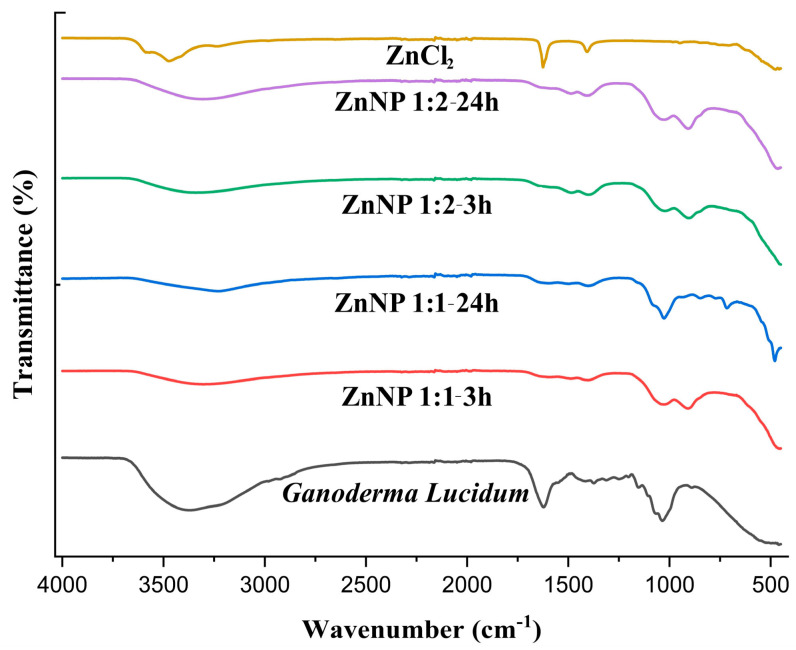
FTIR spectra of ZnCl_2_, Ganoderma Lucidum, and ZnNPs synthesized at different mixing rates (1:1 and 1:2) and times (3 h and 24 h).

The FTIR spectrum of Ganoderma lucidum in Figure 2 show a broad band around 3368.80 due to O-H and C-H. The FTIR spectrum of dried ZnNPs exhibited a small peak at 3305.61, 3232.93, 3307.65, and 3341.33 cm^−1^. This peak was assigned to the -OH stretching vibrations, suggesting the presence of moisture on the surface of the ZnO nanoparticles. The Ganoderma results indicate the presence of water and hydroxyl groups, likely from the high polysaccharide content of the fungal biomass [37]. In contrast, the ZnNPs results suggest the incorporation of hydroxyl groups on the nanoparticle surface, potentially from the green synthesis process using plant extracts [38]. In previous studies, it was mentioned that the FTIR spectrum of Ganoderma lucidum spores showed a band at 1035 cm^−1^, which was attributed to the polysaccharide C-O group [39]. However, the overall structural integrity of Ganoderma lucidum is not directly affected by this specific band because the fungal biomass consists of a complex mixture of polysaccharides, proteins, and other biomolecules. 

The FTIR analysis of the ZnNP samples focuses more on the bands related to the Zn-O bond and the presence of hydroxyl groups, rather than the 1600–1200 cm^−1^ region [13,40]. In one study, the FTIR spectra of ZnO nanoparticles annealed at different temperatures also showed various peaks related to the Zn-O bond in the 400–900 cm^−1^ region. The 400–900 cm^−1^ region is particularly informative for identifying the Zn-O bonds in ZnO nanoparticles [41]. As seen in Figure 2, Zn-O bonds were observed at 909.98, 716.11, 479.38, 907.55, and 902.49 cm^−1^. The lack of significant FTIR peaks in the 3000–3500 cm^−1^ region for pure zinc chloride indicates that the mechanical strength of this inorganic salt is not directly influenced by the presence of hydroxyl or amine groups [42]. The ionic bonding and crystal structure of zinc chloride are the primary determinants of its mechanical properties [43]. In DLS analysis, ZnNPs were subjected to sonication and dissolved in 800 mL of distilled water to obtain a suspension. 

**Figure 3:**
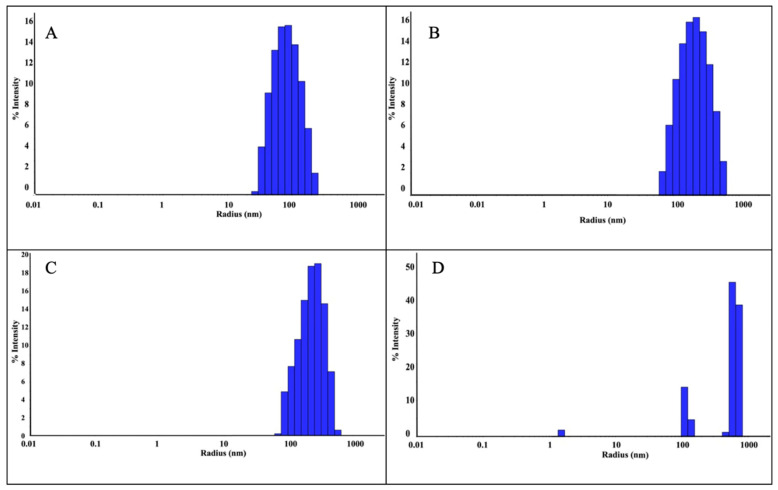
DLS analysis of ZnNPs obtained using mushroom extract/zinc salt, with mixing times of 1:1, 3 h (**A**); 1:1, 24 h (**B**); 1:2, 3 h (**C**); and 1:2, 24 h (**D**).

The size of the ZnNPs obtained after 3 and 24 h of mixing were 90 nm and 243 nm, respectively (Figure 3A,B). As can be seen from the figure, the nanoparticle diameter increased as the mixing time increased. The polydispersity index (PDI) is the ratio of the mass average molecular mass to the number average molecular mass. A low PDI value indicates a narrow distribution and uniformity of molecular weights. In the analysis, the PDI values of the nanoparticles prepared by mixing at a 1:1 ratio were found as 48.69% (Figure 3A) and 51.91% (Figure 3B), respectively. While the increase in mixing time did not change the polydispersity index, it was seen that it caused an increase in nanoparticle size by more than two times. The reason for this can be explained by the increase in agglomeration between nanoparticles as time increases. When the sizes were compared according to 3 h and 24 h mixing times, the size was expected to increase further. However, since the mixing process was also active during this period, the size only reaches this size. It was thought that the size might increase further if they were left stationary in solution. Previous studies in the literature have shown that nanoparticle size can increase depending on mixing time. This shows that the size of nanoparticles increased as a result of the mixing time, which was increased from 3 h to 24 h in this study.

DLS analysis was performed as a result of mixing the nanoparticles synthesized in a 1:2 ratio for 3 h and 24 h. As a result of 3 h of mixing, nanoparticles were detected with a radius of 242.5 nm (Figure 3C). However, as a result of 24 h mixing (Figure 3D), three different peaks were observed, which were attributed to the floral structure [14] seen in the SEM analysis. The PDI value was found to be 43.10% in a 3 h mixture at a 1:2 ratio (Figure 3C). In the 24 h mixture (Figure 3D), PDI values were found in three different values as seen in Table 1. The reason for this situation is attributed to the floral structures seen in the SEM analysis. Since the floral structure was also observed in the previous study [14], the studies are considered consistent. As a result of the experiments, the ZnNPs with the lowest particle size (90.0 nm, PDI 48.69%, 1:1, and 3 h) were selected for use in hydrogel formation. 

The aggregation of nanoparticles during mixing can be attributed to several mechanisms driven primarily by physical and chemical interactions. It is crucial to control nanoparticle size increase as observed in DLS analyses. A phenomenon called Brownian motion and collision can occur between nanoparticles. Brownian motion refers to the random motion of particles suspended in a fluid (liquid or gas) resulting from their collision with fast-moving atoms or molecules in the fluid [44]. This motion pattern consists of random fluctuations in the position of a particle within one subdomain of the fluid followed by its displacement into another subdomain. It is caused by the continuous bombardment of suspended particles by fast-moving atoms or molecules in the fluid. The direction of the atomic bombardment force changes continuously, and at different times the particle is hit harder on one side than the other, resulting in the seemingly random nature of the motion [45].

**Figure 4:**
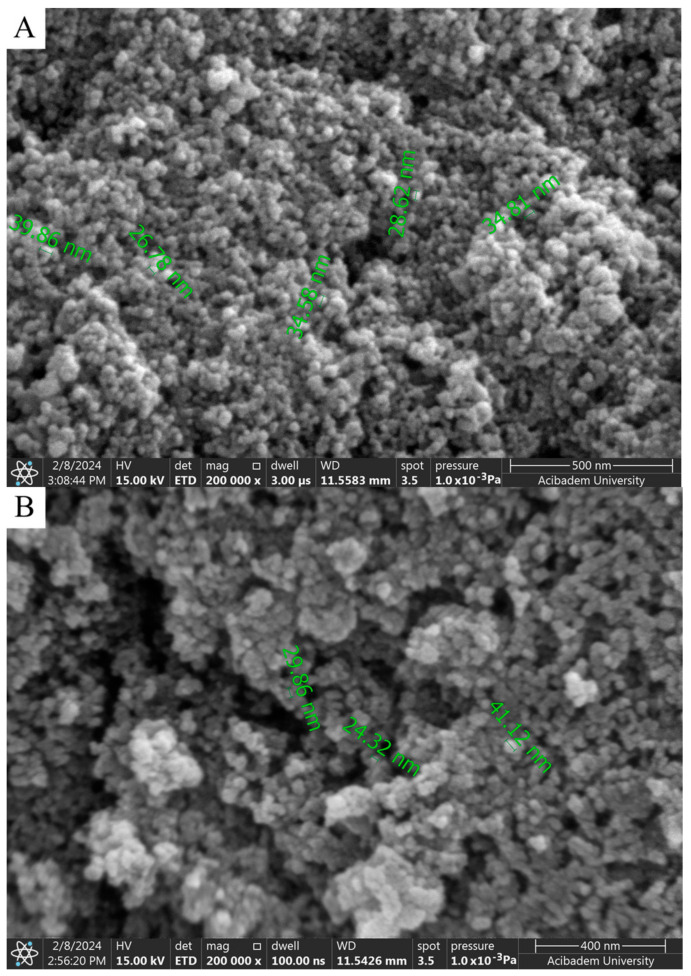
SEM analysis of ZnNPs obtained by mixing mushroom extract/zinc salt (1:1) for 3 h (**A**) and (1:1) for 24 h (**B**) at 200,000× magnification.

**Figure 5:**
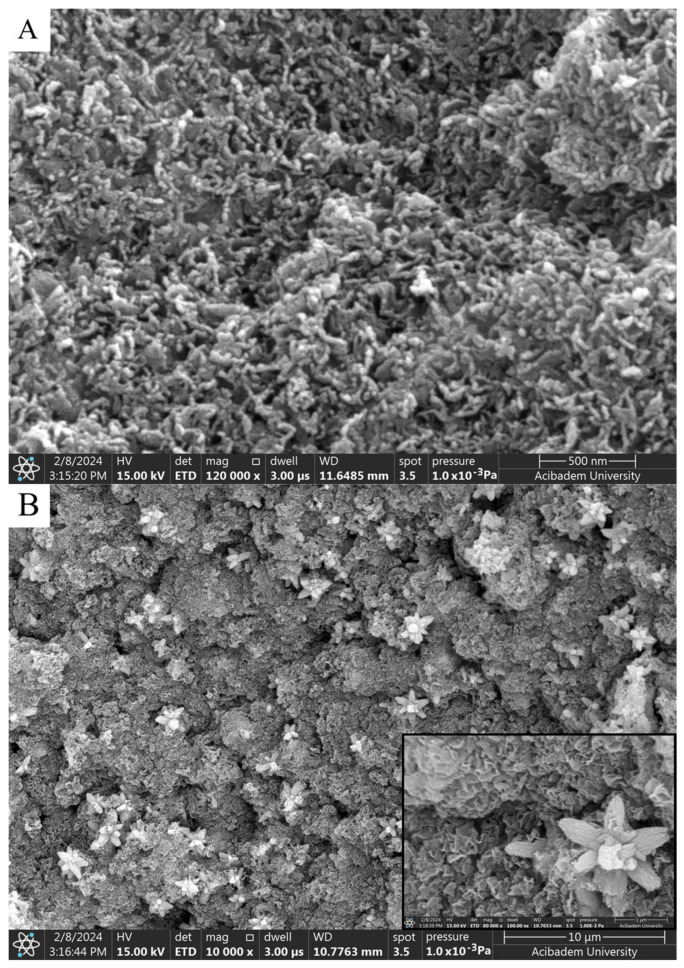
SEM analysis of ZnNPs in mushroom extract/zinc salt (1:2) at 120,000× for 3 h (**A**) and (1:2) at 80,000× for 24 h (**B**).

When SEM images are examined, considering the size analysis in Figure 4A,B, no significant difference was observed between diameters and shapes formed in NPs. However, compared to the nanoparticles synthesized with solutions used in a 1:1 volume ratio, a clear morphological change was observed in the 1:2 synthesis. While the nanoparticles seen in Figure 4A,B exhibited a spherical appearance, those in Figure 5A,B took on a flower-like structure. Morphological differences occurred, which are thought to be due to the mixing difference of the ZnNPs synthesized from zinc salt and mushroom solutions by mixing them in different ratios at different times (Figure 5A,B). In a previous study, Smirnov et al. ZnNPs have been observed to have a flower-like morphological form, and it has been stated that the “petals” of the flower structure of a few hundred nanometers are around 50–200 nm [14]. 

SEM provides a direct image of the particles, measuring their physical dimensions in a dried state. This technique typically yields smaller size estimates as it does not account for surface layers or solvent effects. DLS measures the hydrodynamic diameter of particles based on their Brownian motion in a liquid. The preparation of samples for SEM often involves drying or freezing, which can lead to changes in particle size due to agglomeration or shrinkage [46]. It is believed that this may lead to SEM images showing a size range of 30 to 40 nm. DLS measurements are performed in a liquid medium, and if the particles are prone to agglomeration, the DLS results may reflect the size of these larger aggregates rather than the actual size of the individual nanoparticles [47]. SEM offers a static image of the particles, while DLS gives information about the effective size in solution [46]. 

Aggregation can affect the measured sizes of nanoparticles, leading to differences in the sizes obtained using DLS and SEM. While DLS measures the hydrodynamic diameter of nanoparticles in solution, SEM visualizes the morphology of nanoparticles in the solid state. Therefore, the results obtained from these two techniques can differ.

It is thought that the difference observed in morphology is due to the ratio of solution volumes used. In the nanoparticles obtained after 3 h of mixing, elongated and rod-like structures in contact with each other (Figure 5A) stand out. A flower-like morphological structure of 1–2 microns in size was observed in the nanoparticles obtained as a result of the 24 h mixture (Figure 5B). 

**Figure 6:**
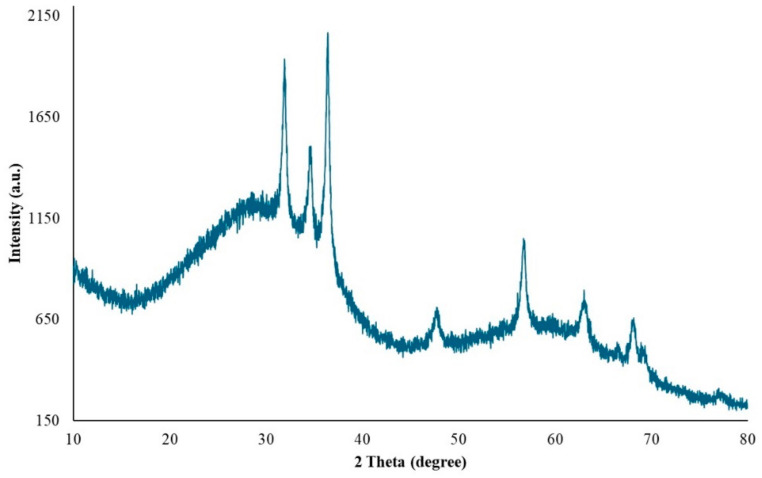
XRD pattern of ZnO nanoparticles.

XRD at 45 kV and 40 mA was used to identify the crystalline phases and estimate the crystallite sizes of the as-prepared ZnNPs. Figure 6 shows the XRD pattern of ZnO nanoparticles obtained by mixing mushroom extract/zinc salt (1:1) for 3 h. In this case, all diffraction peaks at angles of 31.95°, 34.68°, 36.44°, 47.66°, 56.73°, 63.04°, 68.06°, and 69.27° correspond to reflections from the (100), (002), (101), (102), (110), (103), (200), and (201) [48] crystal planes of the hexagonal wurtzite zinc oxide structure. The X-ray diffraction data were recorded by using Cu Kα radiation (1.5406 Angstrom). The intensity data were collected over a 2θ range of 20–80°. The mean size of the ordered ZnNPs was estimated from the full width at half maximum (FWHM) using the Debye–Scherrer formula as follows:

$$D=\frac{K\times \lambda \;}{\beta \times cos(\theta )}$$

where K is the shape factor (usually 0.9), λ is the X-ray wavelength (1.54060 Å for Cu K-α1), β is the line broadening at FWHM in radians, and θ is the diffraction angle. Based on this equation, the mean size of the as-prepared ZnNPs was calculated to be approximately 44.18 nm and no characteristic peaks were observed other than ZnNPs. It is observed that these results are consistent with the SEM findings shown in Figure 4A.

### 2.2. Characterization of Polymer Films 

Films made from PVA and chitosan polymers were produced in two variants: one with ZnNP additives and one without. The films were characterized by SEM, FTIR, and mechanical strength analyses.

**Figure 7:**
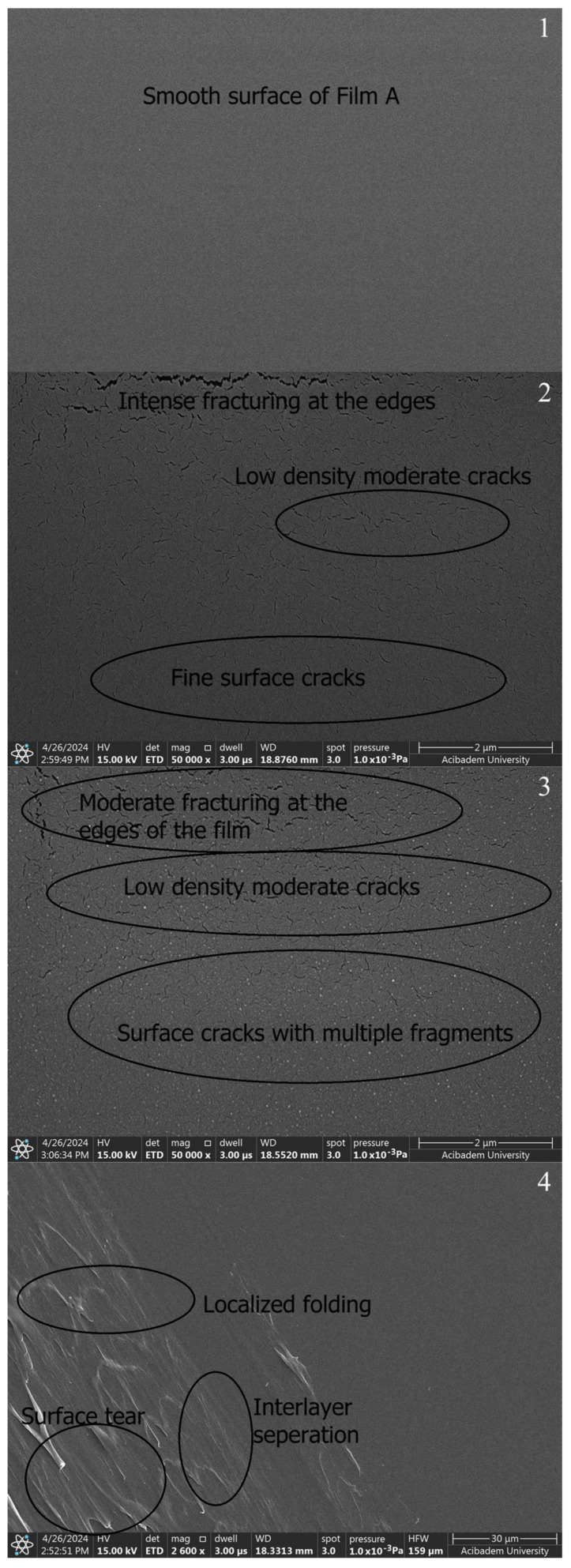
SEM images of polymer films prepared without ZnNPs: A (**1**), B1 (**2**), B2 (**3**), and C (**4**).

When SEM images are examined, smoothness is seen in the films prepared with PVA and Chi polymers (A, B1, and B2) (Figure 7-1–3). The SEM image of the PVA film shows a smooth surface appearance in pure PVA (Figure 7-1) and pure chitosan (Figure 7-2,3). The SEM images reveal that pure PVA exhibits a smooth and flat surface characterized by a homogeneous structure with minimal surface features. This smoothness is an indication of the well-structured nature of the polymer, devoid of significant defects or agglomerations [49]. The PVA/Chi (Figure 7-4) films show a consistent morphology with minimal visible defects. The presence of chitosan contributes to the overall smoothness by increasing the compatibility of the polymeric blend. While pure PVA (Figure 7-1) and pure chitosan (Figure 7-2,3) have a perfectly smooth surface, it is seen that the smoothness starts to deteriorate in the PVA/Chi blend. This is due to the increase in the duration of mechanical mixing stress [50] and thermal conditions [51] in other studies.

As seen in Figure 7-3, it was seen that a layer-like discontinuous structure was formed due to the doubled amount of chitosan. It was observed that there were occasional tears in both chitosan films. It was thought that the reason for this may be due to the electrons sent based on SEM or the freeze–thaw technique applied when preparing those chitosan films. The freeze–thaw technique enhances the porosity and mechanical durability of chitosan hydrogel films, resulting in films suitable for various biomedical and industrial applications [52]. However, this technique can cause tears or mechanical weaknesses in the films. These issues often stem from factors such as rapid freezing and thawing processes, an insufficient homogeneity of the solution, multiple freeze–thaw cycles, and an inadequate use of crosslinkers. To minimize these problems, it is crucial to perform controlled freezing and thawing, thoroughly mix the chitosan solution, optimize the number of freeze–thaw cycles, and use an appropriate amount of crosslinkers. These measures reduce the risk of tearing in the chitosan hydrogel films produced using the freeze–thaw technique, ensuring they possess the desired mechanical and structural properties. Additionally, this technique increases the porosity of the films; ice crystals formed during freezing melt during thawing, leaving behind a porous structure. Multiple freeze–thaw cycles make the porous structure more pronounced. Increased porosity enhances the film’s water retention capacity, expands its surface area, and affects its mechanical properties, which is advantageous for biomedical applications. The mechanical strength of chitosan hydrogel films can be influenced by various factors, including the method of preparation, the degree of crosslinking, and the presence of porosity. In particular, techniques that introduce significant porosity, such as freeze–thaw cycles, can lead to a decrease in mechanical strength due to the formation of voids within the material. Several studies have explored the relationship between porosity and mechanical properties in chitosan hydrogels. For instance, research by Khorasani et al. indicates that while increasing porosity can improve certain functional properties, it can also lead to a reduction in tensile strength and elasticity. The balance between porosity and mechanical strength is crucial for applications in biomedical fields, where both properties are often required [53]. 

**Figure 8:**
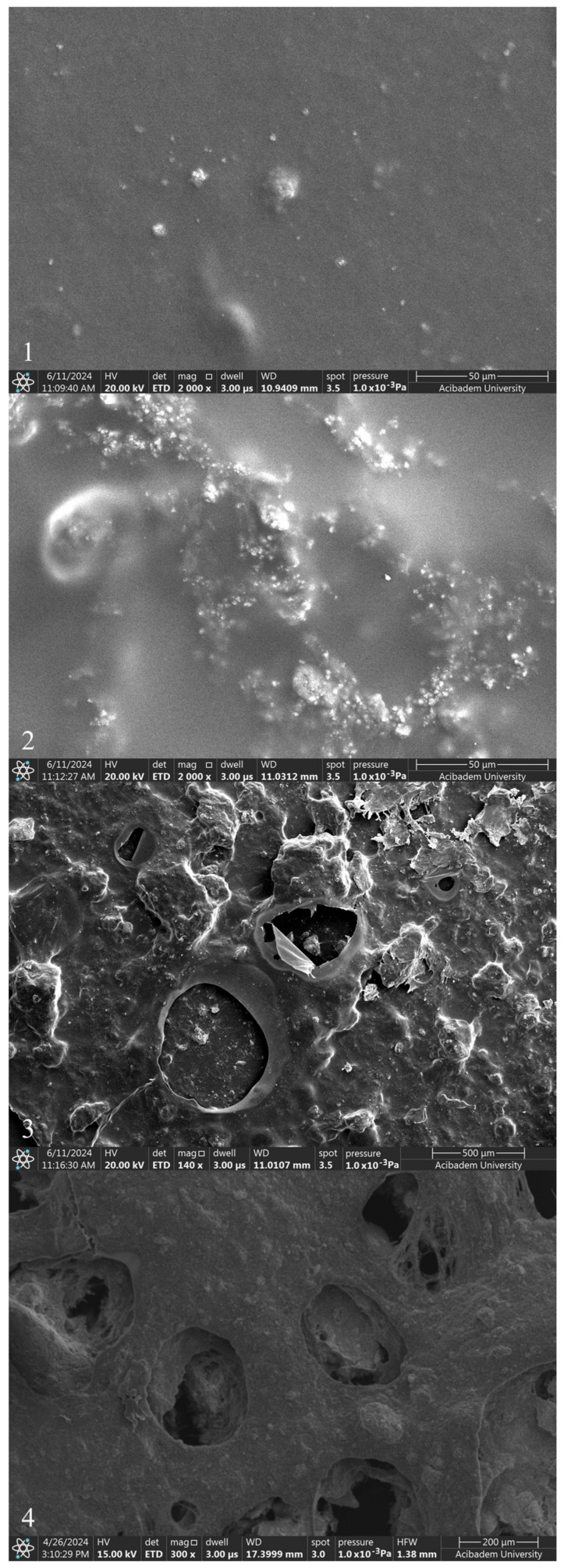
SEM images of polymer films with added ZnNPs: D1 (**1**), D2 (**2**), D3 (**3**), and D4 (**4**).

The incorporation of ZnNPs into PVA/Chi polymer films can significantly alter their properties and behavior. The mixing of ZnO with chitosan and PVA can modify the electrostatic interactions between the polymer components. ZnO interacts with the hydroxyl, amino, and amide groups present in PVA and chitosan, potentially enhancing the mechanical properties of the films. Studies have demonstrated that incorporating 1–5% ZnO can significantly increase both the tensile strength and Young’s modulus, while reducing elongation at break. This indicates strong interactions between the ZnO nanoparticles and the polymer matrix, which constrain the movement of the matrix and increase stiffness [22,54]. However, the presence of pores and cracks observed in the SEM images of PVA/Chi/ZnNPs films could affect their performance. These structural imperfections may influence the interaction between the polymer and ZnO, potentially impacting the mechanical, optical, and other properties of the films [55,56]. While our study focused on the need for such investigations, it specifically examined the mechanical and optical properties. When ZnNPs are added to polymer films prepared using PVA/Chi (C) (Figure 7-4), it has been observed that the smooth structure existing in polymer films begins to deteriorate. The degradation of the structure was associated with the amount of ZnNPs used. No damage was observed in the texture of the ZnNP-doped films which were produced using 50 mg and 100 mg of ZnNPs (D1 and D2) (Figure 8-1,2). As the amount of ZnNPs in the film was increased, tears and air bubble burst marks began to appear in the film (Figure 8-3,4). This causes the films to become more brittle and harder. In the context of ZnNP-doped PVA/Chi polymer films, Van der Waals forces play a significant role in the agglomeration of nanoparticles, particularly as their concentration increases. Additionally, Van der Waals forces between polymer chains can influence the interaction between the nanoparticles and the polymer matrix, affecting the overall distribution and properties of the composite material. Van der Waals forces are weak intermolecular forces that arise from transient dipole moments in molecules and atoms [45,57]. As the concentration of ZnNPs in the PVA/Chi matrix increases, the likelihood of these nanoparticles coming into contact with one another also rises, leading to enhanced Van der Waals interactions. This agglomeration can negatively impact the dispersion of nanoparticles within the polymer matrix, potentially reducing the effectiveness of the nanoparticles in enhancing the mechanical properties of the composite films. To mitigate the effects of Van der Waals forces and minimize agglomeration, strategies such as optimizing the mixing time, using surfactants or stabilizing agents, and employing ultrasonication can be implemented [58,59,60]. 

Biodegradable polymer films containing nanoparticles, such as ZnO, find applications in medical and pharmaceutical fields, where they can serve as protective coatings and drug delivery systems. Additionally, they are utilized in environmental protection for controlled-release systems and antimicrobial surfaces. These films are widely used in food packaging due to their enhanced barrier properties against moisture, oxygen, and UV radiation. ZnNPs are widely favored across various fields for their versatile applications. They are utilized for UV protection, antifungal and antimicrobial effects, photovoltaic experiments, cosmetics, packaging solutions, biomaterials, self-cleaning technologies, environmental sterilization, and as photocatalysts [22]. A significant challenge in integrating metal nanoparticles into polymer matrices is their tendency to agglomerate, which can diminish their photocatalytic and antimicrobial effectiveness and alter the film’s optical, mechanical, and barrier properties. To counteract this, high-speed shearing or ultrasonic techniques are commonly employed to enhance nanoparticle dispersion within the film-forming solutions. The barrier properties of food packaging films are essential, particularly regarding their resistance to water vapor and oxygen permeability. For certain dried food products, preventing moisture absorption from the environment is critical. Additionally, the presence of oxygen can accelerate the growth of food-borne microorganisms, leading to spoilage. However, it is not always practical or desirable for packaging films to completely block oxygen and water vapor. For example, some fresh produce, such as postharvest fruits and vegetables, require a specific amount of oxygen to sustain normal physiological functions during storage. Studies have shown that incorporating ZnNPs into films can enhance their water vapor and oxygen barrier properties, thus improving food preservation. Specifically, the inclusion of 1–5% (*w*/*w*) ZnNPs has been shown to significantly reduce both water vapor and oxygen permeability in films composed of carboxymethyl cellulose, starch-based, chitosan/guar gum, and polyurethane/chitosan composites [54]. In one particular study, researchers developed nanocomposite films by incorporating ZnNPs into a PVA matrix. This combination leverages the natural oxygen barrier properties of PVA alongside the UV-blocking capabilities of ZnNPs, resulting in a film highly suitable for food packaging applications [22]. In another study focused on biomedical applications, ZnNPs were incorporated into an FDA-approved barrier film spray. The nanoparticles were synthesized in three distinct morphologies, and the resulting film was utilized to indirectly protect human fibroblast cells from UV light exposure. The spray, a polymeric solution, formed a uniform, breathable, and transparent film upon application to the skin, which dried rapidly. An SEM analysis of the film revealed aggregates of ZnNPs, with a concentration reaching 10.0 mg/mL. This study aimed to demonstrate the effectiveness of ZnNPs in reducing UV-induced skin damage. Among the tested morphologies, spherical ZnNPs exhibited a higher specific surface area and superior dispersibility compared to other shapes. The inclusion of ZnNPs significantly enhanced the film’s ability to shield damaged skin from UV irradiation, particularly during the wound healing process [55].

**Figure 9:**
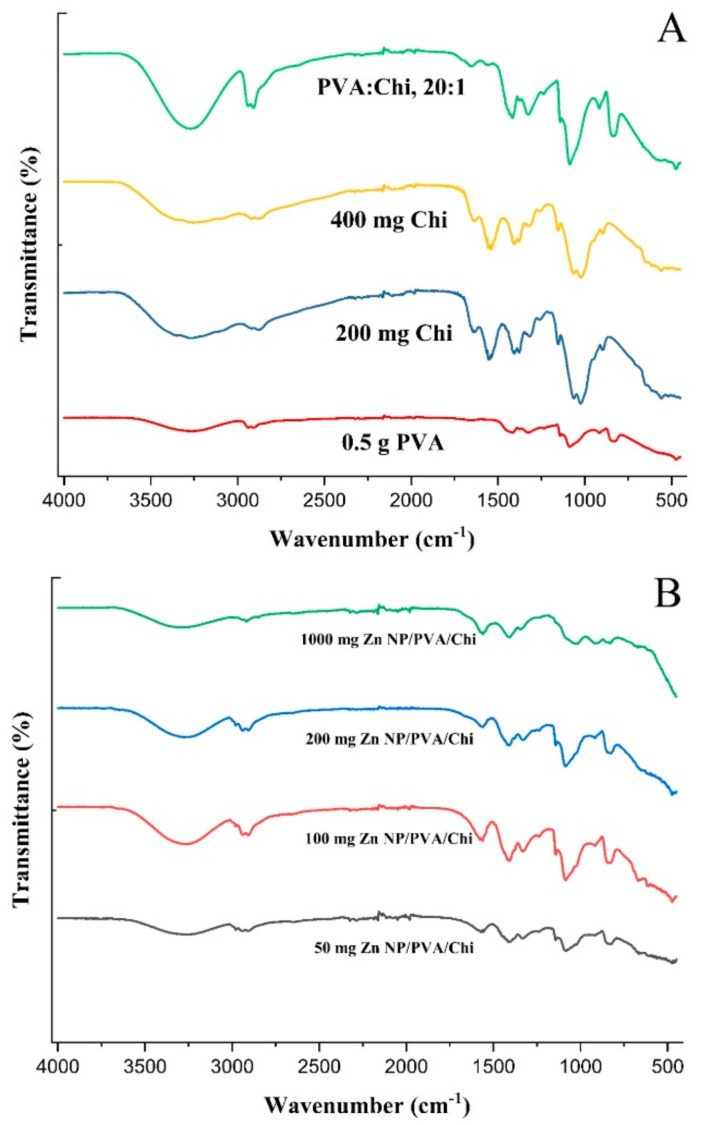
FTIR analysis of polymer films (**A**) and ZnNP-added polymer films (**B**).

The FTIR spectrum of PVA (Figure 9A) typically exhibited strong absorption peaks at around 3300 cm⁻¹, which corresponds to the stretching vibrations of the hydroxyl (-OH) groups. This peak was indicative of the presence of hydrogen bonding between the hydroxyl groups of adjacent PVA chains [61]. Additional peaks at 2900 cm⁻¹ and 1700 cm⁻¹ were attributed to the stretching vibrations of the methylene (-CH_2_-) and carbonyl (-C=O) groups, respectively [62]. Chitosan, a polysaccharide derived from chitin, showed characteristic peaks in its FTIR spectrum. The primary peak was around 3300 cm⁻¹, which was similar to PVA, but it also had a prominent peak at 1650 cm⁻¹, which corresponds to the amide I band (C=O stretching) of the amino groups (-NH_2_). Other notable peaks were methylene stretching at 2900 cm⁻¹ and the bending of -NH_2_ and -CH_2_ groups at 1400 cm⁻¹ [63]. 

When PVA and Chi were blended, the FTIR spectrum showed a combination of the characteristic peaks from both polymers. The hydroxyl (-OH) stretching peak of PVA remains prominent at around 3300 cm⁻¹, while the amide I band of chitosan at 1650 cm⁻¹ was also present. In addition, peaks might appear due to the interactions between the two polymers, such as the formation of hydrogen bonds between the hydroxyl groups of PVA and the amino groups of chitosan [64]. PVA/Chi blends can show evidence of interactions between the two polymers, such as the formation of hydrogen bonds between the hydroxyl groups of PVA and the amino groups of chitosan. These interactions may lead to the appearance of new peaks or changes in the intensity and shape of existing peaks in the FTIR spectrum [65].

The FTIR spectrum of ZnNP-loaded PVA/Chi films (Figure 9B) exhibited a combination of the characteristic peaks observed in the spectra of pure PVA (Figure 9A), pure chitosan, and pure ZnNPs. For example, the strong absorption peak around 3300 cm^−1^ corresponding to the hydroxyl (-OH) groups of chitosan was present, along with the amide I band around 1650 cm^−1^ characteristic of chitosan. Additionally, the ZnNPs contributed their characteristic peaks in the spectrum. The changes in the FTIR spectra of PVA/Chi films with zinc nanoparticles (Figure 9B) indicated enhanced miscibility, the formation of hydrogen bonds, plasticizing effects, and the introduction of new functional groups. These changes collectively contributed to the improved structural integrity of the films, making them more resistant to mechanical stress and environmental factors [65]. 

**Figure 10:**
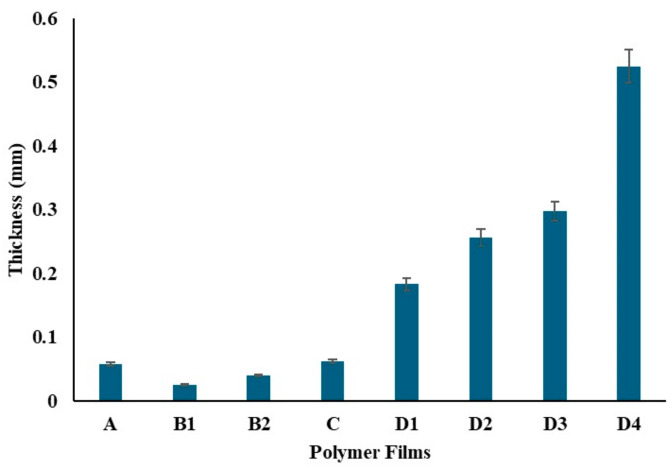
Thickness of the polymer films.

The film thicknesses were evaluated (Figure 10) and analyzed using ANOVA, as presented in Table 1. The analysis revealed a significant effect of different compositions on film thickness (F(7, 16) = 2244.16, *p* < 0.001), indicating substantial differences among the groups. Specifically, Film A, containing only 0.5 g of PVA, showed an average thickness of 0.058 mm. Films B1 and B2, containing 200 mg and 400 mg of chitosan respectively, had thicknesses of 0.025 mm and 0.04 mm. The Tukey HSD test confirmed significant differences between these films. Film C, with both 0.5 g PVA and 25 mg chitosan, displayed an average thickness of 0.062 mm, which was not significantly different from Film A. Films D1, D2, D3, and D4, which included increasing amounts of ZnNPs, exhibited progressively higher thicknesses, with the most significant differences observed for Film D4 (1000 mg ZnNP).

To determine the tensile strength of the produced films, PVA, Chi, PVA/Chi, and ZnNP-doped PVA/Chi films were cut into rectangular shapes of 1 cm × 4 cm, attached between the compression arms of the device, and pulled at a rate of 5 mm/min. Table 2 shows the thickness of the produced biopolymer films measured with the help of calipers before tensile-rupture analysis. 

**Figure 11:**
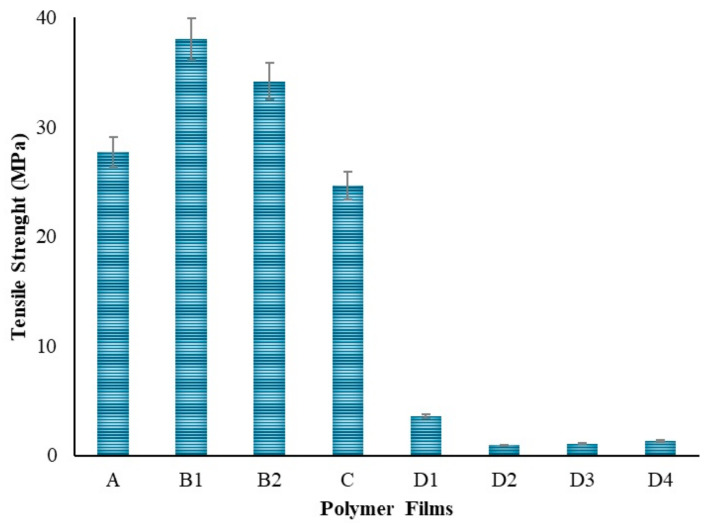
Tensile strength of the polymers.

PVA is a polymer known to have an elastic structure. Hydrogen bonding between the hydroxyl groups of PVA molecules plays a crucial role in its elastic properties [66]. Additionally, water reduces the mechanical strength but increases the plasticity of PVA, making it more elastic [67]. With the addition of chitosan to the PVA polymer as seen in Figure 11, a decrease in tensile strength and elongation at break was observed. This result is consistent with the literature [68]. This indicates a decrease in the flexibility and elasticity of the film. Hydrogen bonds [66] and charge interactions [68] may be among the reasons for the decrease in strength when chitosan is bonded to PVA. In the strength analysis, it was seen that the polymer film with the highest elongation is the PVA film with 42 mm. When the Chi polymer is added to the PVA, the strength of the film decreases due to the natural brittleness [69] of chitosan. In addition, the molecular weight of chitosan plays an important role. Chitosan with lower molecular weight has less tendency to entanglement and interaction with PVA, which may lead to a decrease in the tensile strength and elongation properties of the mixture [58]. It is thought that using low molecular weight chitosan in this study might be one of the factors in the decrease in strength. In polymer films prepared by adding 50–100–200 mg of ZnNPs, a shape change occurred in the polymer films due to the increase in ZnNPs. It is thought that this is due to the hardness created by the ZnNPs added to the film. The lowest strength was observed in the polymer film prepared by adding 1000 mg. Depending on the amount of ZnNPs used, holes formed in the film and caused it to become a brittle structure. There might be multiple reasons for the decrease in tensile strength: nanoparticle agglomeration [70], poor interfacial bonding [70], and concentration effects [58].

**Figure 12:**
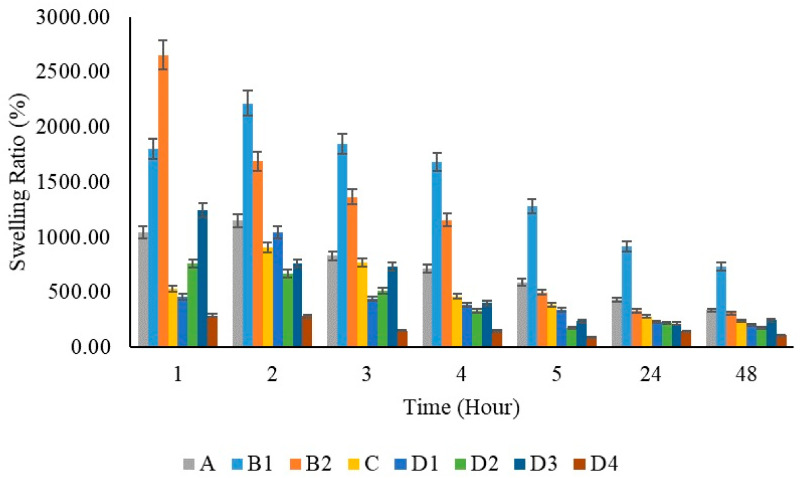
Swelling capacity graphs of polymer films per hour.

Polymeric substances have the capability to expand and hold a considerable volume of water within their framework. They maintain a flexibility level akin to natural tissue because of their high water content. Hydrogels’ water absorption ability is due to hydrophilic groups attached to the polymer backbone, while their resistance to dissolving is due to cross-links among network chains [71]. When PVA films were immersed in PBS solution, the swelling ratio was reported to be 674% in previous studies. In Figure 12, a lower swelling capacity was observed in the pure PVA film compared to the pure chitosan films at the first hour. When the chitosan films were evaluated at different concentrations within themselves (B1 and B2), it was observed that the swelling capacity increased as the amount of chitosan increased. The swelling capacity of the film prepared using 200 mg chitosan increased by 1800% at the first hour; the swelling capacity of the film prepared using 400 mg chitosan increased by 2655% at the first hour.

The immersion procedure in PBS [72] was applied as specified by literature review for swelling testing. Hydrogels exhibit different swelling ratios in PBS at various temperatures. At room temperature (approximately 20–25 °C), the swelling ratios may be lower compared to those observed at physiological temperatures (37 °C) due to reduced kinetic energy and slower diffusion rates of water into the hydrogel matrix [73,74]. Based on this information, swelling tests of hydrogels were performed at room temperature, 25 °C. Numerous studies have demonstrated that in PVA/Chi blends, swelling at pH 7 is significantly lower compared to other pH levels [30,75,76]. One example of this is a study where it was observed that PVA/Chi hydrogels exhibited a reduced swelling ratio at pH 7, which was notably lower than the swelling observed at both acidic and alkaline pH conditions. In that study, it was found that the hydrogel’s pH sensitivity is linked to the different swelling behaviors observed in buffer solutions with varying pH levels. The CS/PVA hydrogel exhibited reduced swelling ratios at pH 7, 10, and 13 when compared to those at pH 1 and 4. Interestingly, the swelling degree at pH 1 nearly doubled relative to pH 13. This phenomenon was attributed to the ionic strength of the solution, where chitosan facilitated more effective crosslinking, both chemically and physically, creating a 3D network structure. This enhanced entanglement decreased repulsive forces and improved intramolecular interactions, especially at pH 5.5–6 in the PVA/Chi hydrogel blend. At acidic pH levels, the amino groups of chitosan became protonated, leading to electrostatic repulsions that increased the hydrogel’s hydrophilicity, thereby expanding its network. Conversely, at neutral (pH 7) and alkaline (pH 10 and 13) conditions, the deprotonation of these amino groups resulted in a reduced swelling capacity [75]. Therefore, the study suggested that the pH-sensitive swelling behavior of the hydrogel, particularly its reduced swelling at neutral pH, could be advantageous for controlled and sustained drug release, meeting the increasing demand for high-accuracy drug delivery systems. At pH 7, the hydrogel structure is characterized by the deprotonation of amino groups, leading to a decrease in swelling capacity. This reduced swelling results in a tighter hydrogel network, which in turn slows down the release of encapsulated drug molecules. Consequently, at neutral pH, the hydrogel may provide a more controlled and sustained release profile, which could be advantageous in applications requiring prolonged drug delivery. However, this slower release rate might be less desirable in scenarios where rapid drug release is necessary.

It was stated that the swelling capacity in pure chitosan increased by 1.5 times when the concentration was doubled. It is known that the reason for this increase is due to the hydrophilicity of chitosan [77]. However, it was observed that the swelling capacity decreased by half when chitosan was added to PVA films. This is thought to be due to the excessive use of PVA at a ratio of 20:1. In similar studies [78], a decrease in the water absorption capacity of the PVA/Chi composite was observed as the amount of PVA increased. All films showed maximum swelling capacity at 2 h. With the addition of ZnNPs to PVA/Chi films, swelling capacities increased 1.6 times among the films prepared by adding 50, 100, and 200 mg of zinc depending on the zinc ratios. However, in the film prepared using 1000 mg of ZnNPs, the absorption capacity decreased due to the excess amount of zinc. At the 24 h mark, the addition of zinc nanoparticles at concentrations of 50, 100, and 200 mg did not result in significant changes in swelling percentage. However, when the amount of added zinc nanoparticles was increased to 1000 mg, a more pronounced reduction in swelling capacity was observed. ZnNPs can cause a decrease in the swelling capacity of hydrogels for several reasons: Increased cross-linking density may make the polymer network tighter, reducing water absorption. Nanoparticles can alter the microstructure of the hydrogel by filling the voids between polymer chains, making it harder for water to penetrate these spaces. Surface interactions can be affected, as ZnNPs might reduce the hydrophilic interactions between the hydrogel and water, leading to decreased swelling capacity. Additionally, nanoparticles may increase the overall rigidity of the hydrogel, which reduces its flexibility. Aggregation issues can also occur if nanoparticles do not disperse evenly, preventing water from spreading throughout the gel.

At equilibrium swelling, the swelling ratio is governed by the balance between osmotic pressure and network elasticity. Recent advances in hydrogel design, aimed at achieving optimal mechanical performance, have been comprehensively reviewed in several notable papers, especially those focusing on polymer network structures and nonlinear elastic fracture mechanics. Considering the significance of swelling and deswelling behavior in hydrogels and the practical necessity for hydrogels to reach an equilibrium swelling state in a liquid medium, we begin with a basic hydrogel model to elucidate the effects of molecular structure and swelling or deswelling on mechanical properties [79]. The amount of water such structures can hold at equilibrium varies based on the polymer’s properties and the nature and density of the network junctions. Typically, in the swollen state, hydrogels contain a much higher mass fraction of water compared to the polymer. Hydrogels, as three-dimensional crosslinked hydrophilic polymer networks, can reversibly swell or de-swell in water and retain a large volume of liquid when swollen. The degree of swelling depends on both the doses and concentrations due to changes in the cross-linking density within the hydrogels. Most volume change in hydrogels during swelling happens within the first 24 h. The swelling rate of superabsorbent polymers can range from a fraction of a minute to several hours. Rapid swelling in those polymers is primarily due to their small sample size [71]. In all synthesized films, a high absorption capacity was observed in the first three hours. After the third hour, the films gradually switched to release mode. When looking at the 24 h, it is seen that adding 50–100–200 mg of ZnNPs to the system does not cause significant changes in the swelling ratio percentage, while increasing the amount of added zinc nanoparticles to 1000 mg causes a greater decrease in the swelling capacity.

The presence of pores in the films did not significantly impact the swelling capacity up to a certain extent. However, the D3 film exhibited slight tearing, while the D4 film showed more extensive tearing across multiple layers, leading to the appearance of pores. Despite these observations, the roughness of the films did not negatively affect their swelling capacity until the addition of 1000 mg of ZnNPs. Notably, the polymer film containing 200 mg of ZnNPs (D3) demonstrated a greater swelling capacity than the other ZnNPs loaded films (D1, D2, and D4). The increase in ZnNPs concentration enhanced the swelling capacity, facilitating greater liquid absorption. This finding aligns with previous studies, which have similarly reported that an increase in ZnNP concentration within polymer films typically results in a higher swelling ratio [9]. This phenomenon can be attributed to the increased hydrophilicity associated with a higher nanoparticle content, which creates additional surface area for water absorption. Specifically, the film containing 50 mg of ZnNPs (D1) exhibited a swelling ratio of approximately 500%, while the film with 100 mg (D2) showed a swelling ratio of approximately 750%. When the concentration was further increased to 200 mg (D3), the swelling ratio reached a maximum of approximately 1250%. This trend is consistent with prior research. However, at a concentration of 1000 mg, the swelling capacity decreased significantly to approximately 250%. Studies on the swelling properties of polymeric materials, especially hydrogels, have shown that increasing the concentration of nanoparticles such as ZnNPs increases their swelling capacity and liquid absorption. Increasing the ZnNPs concentration increases the swelling capacity of hydrogels, allowing for greater liquid absorption. Studies have consistently reported that an increase in the ZnNPs concentration within polymer films generally results in a higher swelling ratio [80,81].

In the context of PVA/Chi films, it was observed that tensile strength increased with the addition of ZnNPs at concentrations of 1–5% [82]. However, in the experimental design of this study, the concentration added to the polymer films was 9.5%. Literature indicated that concentrations in the range of 1–5% enhanced both tensile strength and flexibility. In contrast, concentrations around 10% could lead to nanoparticle clustering. At such levels, while the nanoparticles were expected to distribute throughout the polymer matrix, excessive ZnNPs could aggregate, reducing flexibility and making the film more brittle. Elongation at break, which measures the film’s ability to stretch before breaking, initially increased with ZnNPs addition. However, at higher concentrations (e.g., 10%), this property decreased due to nanoparticle agglomeration, leading to brittleness and reduced flexibility [83]. Such agglomeration created weak points in the film structure, diminishing overall integrity. Young’s modulus, reflecting the stiffness of the films, increased with higher ZnNPs concentrations, but this increase came at the expense of flexibility, making the films more brittle and less able to absorb energy [84]. In the experiment, polymer films containing ZnNPs at concentrations higher than those reported in the literature showed increased susceptibility to breakage. The lowest tensile strength observed in films with 100 mg of ZnNPs was attributed to nanoparticle agglomeration at this higher concentration. Conversely, films containing 50 mg of ZnNPs exhibited the highest tensile strength due to better nanoparticle dispersion within the polymer matrix. Although higher concentrations (200 mg and 1000 mg) improved tensile strength compared to the 100 mg films, they did not surpass the performance of the 50 mg films. Mechanical strength decreased at concentrations of 9.5% and above. Swelling tests indicated that films prepared with 50–100–200 mg of ZnNPs swelled in accordance with their concentrations during the first hour. By the second hour, the film with 50 mg of ZnNPs continued to swell, while those with higher concentrations began to release. At the 24th hour, swelling was minimal compared to the 5th hour. The film containing 1000 mg of ZnNPs exhibited slower swelling and release due to the high amount of nanoparticles. The films with 50–100–200 mg of ZnNPs (D1-2-3) demonstrated controlled release and shrinkage behavior. However, extremely high concentrations led to complex results, despite the constant environmental conditions (pH, temperature, mixing time, and speed).

## 3. Conclusions

This study successfully demonstrated the green synthesis of zinc nanoparticles using Ganoderma Lucidum mushroom extract, marking a novel approach in nanoparticle production. The synthesis process was characterized by DLS, SEM, XRD, and FTIR spectroscopy, which revealed that the nanoparticle yield and size distribution were significantly influenced by the extract/salt ratio and mixing duration. Longer mixing times led to increased nanoparticle size and polydispersity index, attributed to agglomeration phenomena. The incorporation of zinc nanoparticles into polymer films made of PVA and chitosan showed distinct effects on their structural and mechanical properties. Chi addition resulted in reduced tensile strength and the elongation of PVA films, while excessive ZnNPs led to increased brittleness and structural degradation. Swelling capacity tests further demonstrated that pure chitosan films exhibited higher swelling compared to pure PVA films, with an initial enhancement in swelling capacity observed with ZnNPs, though excessive nanoparticles led to decreased swelling due to increased cross-linking and aggregation.

The Tukey HSD test results highlight that statistically significant differences exist between almost all group pairs, with *p*-values very close to zero, indicating that these differences are highly significant. The extremely small *p*-values, such as approximately 0.00019792 for A vs. B1 and 1.55 × 10^−15^ for A vs. D4, confirm the robustness of the findings. In contrast, the *p*-value for A vs. C was approximately 0.99241, suggesting no significant difference between these groups. The observed variations in film thicknesses underscore the significant impact of ZnNP and chitosan concentrations. Overall, the findings highlight the critical role of synthesis conditions and polymer composition in determining the properties of nanoparticle-filled polymer films. This research provides foundational insights for future developments in wound dressing materials and other applications requiring functionalized nanocomposites. The innovative use of Ganoderma Lucidum extract for the green synthesis of zinc nanoparticles opens new avenues for sustainable nanotechnology practices.

## 4. Materials and Methods

### 4.1. Extraction of Ganoderma Lucidum Mushroom

Dried Ganoderma lucidum mushrooms were obtained from an herbalist. The mushroom bodies provided were structurally examined by FTIR spectroscopy. Stem pieces were weighed to 10 g and boiled in 150 mL of distilled water for 3 min. Afterwards, the extract was allowed to cool and separated from the solid components by filtering with filter paper (Whatman no: 1) and kept at +4 °C until used [13].

### 4.2. Green Synthesis of ZnNPs

A total of 1 g of zinc chloride (Zag Kimya, ≥98.00% purity) was dissolved in 100 mL of distilled water and the pH of the solution was 6.73. Different ratios of mushroom extract and zinc solution (1:1, 1:2) were mixed for 3 h [85] and 24 h [86] to obtain the optimum nanoparticle. The extract and solution were mixed by using an ultrasonic homogenizer (Bandelin Ultrasonic Homogenizer HD 2070.2, Berlin, Germany, Probe: MS 73, Hm: 60%, pulse on 10 s off 2 s) for 4.5 min. After, the pH value of the mixture was adjusted to 12 with 0.1 M NaOH. The mixture was centrifuged at 10,000 rpm for 12 min and then washed with distilled water and ethanol. Following that, the solid phase was dried in an incubator at 60 °C. Dried samples were stored at +4 °C in a desiccator until next use.

### 4.3. Preparation of Pure PVA, Pure Chi, and PVA/Chi Films

A total of 0.5 g of PVA (Sigma-Aldrich, Upper Bavaria, Germany) was dissolved in 5 mL of distilled water by stirring in a magnetic stirrer at 70 °C [87]. Totals of 25, 50, 100, 200, and 400 mg of Chi (Sigma-Aldrich) were dissolved in 1% acetic acid solution [88] in separate beakers. After the mixing process was completed, Chi was poured into the PVA polymer solution at room temperature. A 1100 rpm stirring speed at 30 min was applied to all solution preparation procedures. PVA, Chi, and PVA/Chi films were prepared for characterization. Pure PVA and pure chitosan films were prepared in order to make more accurate comparisons between the films. Pure polymer films were formed by dissolving 0.5 g of PVA in 5 mL of distilled water and 25–50–100–200–400 mg of chitosan in 1% acetic acid solution using the same procedure as the PVA/Chi film. Since the films prepared using 25–50–100 mg of pure chitosan were too thin and could not provide integrity, films produced using 200 and 400 mg of chitosan were used in the experiment.

### 4.4. Preparation of ZnNP-Doped Polymer Films

Films were prepared by mixing polymers and solid ZnNP powder in the specified proportions (Table 3) by using a magnetic stirrer for 30 min at 1100 rpm, then kept in the sonicator at room temperature for 30 min. Then polymer hydrogel films were left to dry in the oven at 60 °C.

### 4.5. Characterization of ZnNPs and Hydrogel Films

#### 4.5.1. DLS Analysis

DLS is a very powerful tool for studying the diffusion behavior of macromolecules in solution. The diffusion coefficient and hence the hydrodynamic radius calculated from it depend on the size and shape of the macromolecules. The radius of ZnNPs was measured with the DynaPro NanoStar DLS device (Wyatt Technology, Santa Barbara, CA, USA).

#### 4.5.2. XRD Spectroscopy Analysis

The crystallographic structure and chemical composition of the ZnNPs were determined using X-ray diffractometry. The analysis was conducted with a Malvern PANalytical X’Pert PRO device (Worcestershire, England), where the samples, prepared in powder form, were irradiated with X-rays. The diffractometer scanned the samples over a 2θ range of 20° to 80°, allowing for detailed information on both the chemical composition and crystal structure of the ZnNPs to be obtained.

#### 4.5.3. SEM Analysis

A morphological analysis of ZnNPs and polymer films (PVA, Chi, PVA/Chi and PVA/Chi/ZnNPs) was carried out with a scanning electron microscope (Thermo Scientific Quattro S, Thermo Fisher Scientific, Brno, Czech Republic). SEM is a powerful magnification tool that uses focused beams of electrons to obtain information. It provides detailed surface data for solid samples [89]. In this study, a gold-coating process was applied using a Leica EM ACE200 vacuum coater.

#### 4.5.4. FTIR Spectroscopy Analysis

Vibrations in the mid-infrared range originate from many environmentally important molecules such as organic acids, soil organic matter, mineral phases, and oxyanions. In the mid-IR range (400–4000 cm^−1^), there are several extremely important and useful classes of bonds that can be studied. Within the scope of this study, the chemical characterization of ZnNPs and polymer films (1 × 1 cm^2^) was carried out using the FTIR analysis method in the 450–4000 cm^−1^ wave range.

#### 4.5.5. Tensile-Rupture Strength Analysis

A Shimadzu AGS-X brand testing machine was used to conduct strength analyses of wound dressing films. First, the thickness of the polymeric films was measured with the help of calipers. The films were cut to lengths of 1–4 cm, with the initial length $L_{0}$ set to 2 cm, and the films were secured from their ends. The stretching speed of the films was set to 5 mm/min. Subsequently, the tensile-rupture strength analysis was carried out using a 5 kN load cell. Tensile tests are often conducted at room temperature, which is suitable for many polymer-based materials, including those containing metallic nanoparticles. Testing at room temperature allows for the assessment of mechanical properties without the influence of thermal effects that could alter the results [90]. Based on literature review information, strength analysis was carried out at 25 °C and 52% humidity.

#### 4.5.6. Swelling Analysis

The swelling efficiency of the hydrogel is closely linked to its microstructure and porosity. For the swelling test, samples were taken from the prepared films, and their dry weights were recorded as Wk. Each cut hydrogel film (circles with a diameter of 0.5 cm) was then separately added to 5 mL of PBS buffer solution. The films were allowed to swell and were subjected to swelling analysis at room temperature for 48 h. Weight measurements of the swollen hydrogels were taken at intervals of 1, 2, 3, 4, 5, 24, and 48 h with their masses recorded as Ws. The swelling behavior of hydrogels containing varying amounts of PVA, Chi, and ZnNPs were determined, and the swelling ratio equations were calculated using Equation (1) [91]. (Ws: the weight of swollen hydrogel (mg). Wk: the weight of dry hydrogel (mg)).

(1) $$\mathrm{Percent}\;\mathrm{Swelling}\;\mathrm{Rate}\;(\%\mathrm{PSR})=\frac{Ws-Wk}{Wk}\;\times \;100$$

#### 4.5.7. Statistical Analysis

In this study, statistical analyses were conducted to evaluate the effects of different film compositions on thickness. Statistical analysis data were expressed as mean ± SD. Variance analysis (ANOVA) was employed to determine if there were significant differences in the average thicknesses among the various film groups. The results of the ANOVA test were reported using F-statistics and *p*-values. When ANOVA indicated significant differences, post-hoc analysis was performed using the Tukey HSD (Honestly Significant Difference) test to identify which specific group pairs exhibited significant differences. The *p*-values, which were assessed against a significance level of 0.05, determined the statistical significance of the differences between groups. All statistical analyses were performed using GraphPad Prism, and the results were interpreted based on the calculated *p*-values and F-statistics. A *p*-value less than 0.05 (*p* < 0.05) was accepted as significant in both tests.

## Figures and Tables

**Table 1:** DLS analysis of ZnNPs according to mixing rate/hour.

Extract/ZnCl_2_ Ratio (*V*:*V*), Hour	Sizes of ZnNPs (nm)	PDI (%)
(1:1), 3 h	90.0	48.69
(1:1), 24 h	243.3	51.91
(1:2), 3 h	242.3	43.19
(1:2), 24 h	1.5; 117.4; 647.9	2.72; 10.97, 12.43

**Table 2:** Thickness of polymer films.

Polymer Films								
	A	B1	B2	C	D1	D2	D3	D4
Thickness (mm)	0.058 ± 0.003	0.025 ± 0.001	0.040 ± 0.002	0.062 ± 0.003	0.183 ± 0.009	0.256 ± 0.013	0.297 ± 0.015	0.525 ± 0.026

**Table 3:** Amounts of components in the hydrogel films.

Films	Film Codes	PVA (g)	Chi (mg)	ZnNPs (mg)
PVA	A	0.5	0	0
Chi	B1	0	200	0
Chi	B2	0	400	0
PVA/Chi	C	0.5	25	0
PVA/Chi/ZnNPs	D1	0.5	25	50
PVA/Chi/ZnNPs	D2	0.5	25	100
PVA/Chi/ZnNPs	D3	0.5	25	200
PVA/Chi/ZnNPs	D4	0.5	25	1000

## Data Availability

The raw data supporting the conclusions of this article will be made available by the authors on request.
